# A Challenging Case of Genetically and Histologically Diagnosed Pulmonary Veno-Occlusive Disease with Extracorporeal Life Support and Redo Lung Transplantation

**DOI:** 10.1155/2023/4846338

**Published:** 2023-08-22

**Authors:** Mohamed Laimoud, Ziyad Alanazi, Fayez Alahmadi, Abdullah Aldalaan

**Affiliations:** ^1^Cardiac Surgery Critical Care Department, King Faisal Specialist Hospital and Research Center, Riyadh, Saudi Arabia; ^2^Critical Care Medicine Department, Cairo University, Cairo, Egypt; ^3^Royal College of Surgeons in Ireland, Dublin, Ireland; ^4^Pulmonary Medicine Department, King Faisal Specialist Hospital and Research Center, Riyadh, Saudi Arabia

## Abstract

**Background:**

Pulmonary veno-occlusive disease (PVOD) is a rare form of pulmonary arterial hypertension characterized by diffuse venous vasculopathy and increased pulmonary vascular resistance resulting in right-sided heart failure. *Case Presentation*. A 22-year-old female patient started to have dyspnea with minimal effort and was diagnosed to have pre-capillary pulmonary hypertension (PH) with right-sided heart failure. Initially, she was diagnosed to have idiopathic PH. She developed life-threatening pulmonary oedema and cardiogenic shock after pulmonary vasodilator therapy. A genetic study was done and revealed the eukaryotic translation initiation factor 2 alpha kinase 4 (EIF2AK4) gene on chromosome 15, which was diagnostic to heritable PVOD. After failure to achieve hemodynamic stabilization with conventional cardiopulmonary support measures, extracorporeal membrane oxygenation (ECMO) supported her till bilateral lung transplantation, which was unfortunately complicated by acute graft rejection. After a prolonged intensive care unit stay with 4-month ECMO support, the second bilateral lung transplantation was done, and the patient survived and was discharged.

**Conclusions:**

Clinical recognition of PVOD is crucial due to its challenging diagnosis, need for genetic study, rapid deterioration with pulmonary vasodilators, and bad prognosis. Lung transplantation is the definitive treatment for eligible candidates.

## 1. Background

Pulmonary hypertension (PH) is defined as a mean pulmonary arterial pressure (mPAP) ≥20 mmHg at rest, whereas the pre-capillary PH includes mPAP ≥20 mmHg, pulmonary capillary wedge pressure (PCWP) ≤15 mmHg, and pulmonary vascular resistance (PVR) <2 Wood units (WU) [1]. Pulmonary arterial hypertension (PAH) is related to pre-capillary aetiology as classified by the World Health Organization [1–3]. The prevalence of PAH is 48–55 cases/million adults, mostly females [1, 4]. The accurate prevalence of pulmonary veno-occlusive disease (PVOD) is difficult to be estimated due to difficulty in diagnosis without genetic and histopathological studies and many cases usually being treated as idiopathic PAH. It was suggested that PVOD may account for one to two cases per million inhabitants based on the histopathological series of patients labelled as idiopathic PAH [5]. Unlike idiopathic PAH, PVOD can affect all age groups and both sexes equally with autosomal recessive transmission [6]. We report a sporadic case of PVOD with a challenging journey from diagnosis until hospital discharge after prolonged extracorporeal membrane oxygenation (ECMO) and repeated bilateral lung transplantation.

## 2. Case Presentation

A 22-year-old female patient with a body mass index (BMI) of 20.6 kg/m^2^, started to complain of progressive shortness of breath with minimal efforts without other symptoms after delivery of her first kid with uncomplicated pregnancy. She did not have a history of abortion or a family history of cardiac or pulmonary disease. She presented to the emergency department after 4 months of hospital discharge. Initial evaluation showed sinus tachycardia, jugular venous distension, systemic blood pressure (BP) 105/65 mmHg, pulse oximetry saturation 86% on ambient air, bilateral lower limbs mild pitting oedema, and a loud pulmonary component of the second heart sound. Laboratory workup revealed: haemoglobin 117 (g/L), platelets count 180 (×10^9^/L), white blood cells 10 (×10^9^/L), serum Na 134 mmol/L, NT-proBNP 500 (pg/mL), normal kidney, and liver chemistries. Septic and virology screening was done and was negative for human immunodeficiency virus and hepatitis B and C antibodies. Screening for Schistosomiasis antibodies was negative. An autoimmune profile was done and revealed negative for antinuclear antibody, anti-neutrophil cytoplasmic autoantibody, lupus anticoagulant, anti-Sjögren's syndrome-related antigen A autoantibodies, and anti-SS-antigen B autoantibodies antibodies. Chest X-ray (CXR) was unremarkable. Ventilation/perfusion (V/Q) scan excluded chronic thromboembolic pulmonary hypertension. Pulmonary function tests (PFTs) were done and showed that the forced expiratory volume (FEV1) was 1.3 (L), and the forced vital capacity was 1.5 (L).

**Figure 1:**
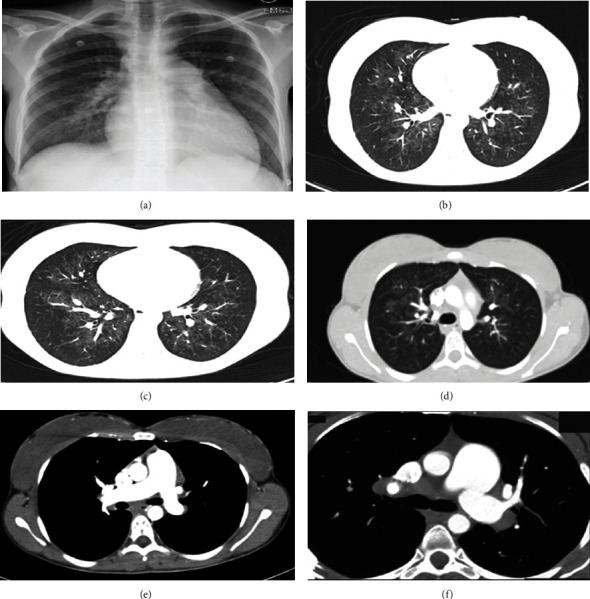
Admission CXR (a) showing clear lung fields and normal cardiac silhouette. High-resolution CT chest scan (b–f) showing bilateral peri-bronchovascular ground glass densities involving upper and lower lobes with smooth interlobar septal thickening, mediastinal lymphadenopathy, and pulmonary trunk dilatation.

**Figure 2:**
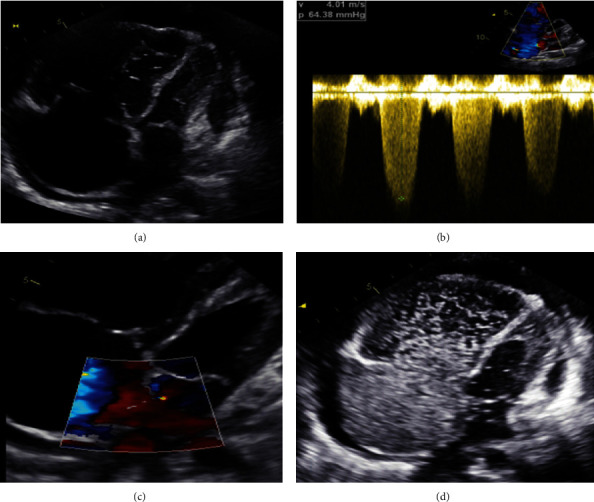
Transthoracic echocardiography revealed severe dilatation and systolic dysfunction of the right ventricle while normal left ventricle and moderate pericardial effusion (a). There was severe TR with an estimated PASP >80 mmHg and TRV 4.01 m/s (b). The right atrium was severely dilated with a small atrial septal defect (c). TEE revealed a positive agitated saline test confirming interatrial shunting (d).

Transthoracic echocardiography (TTE) revealed severe right ventricle dilatation with moderately reduced function, normal-sized left ventricle with ejection fraction (EF) of 50–55%, and flattened interventricular septum. The right atrium was severely dilated with a small atrial septal defect (ASD) above the fossa ovale, there was a severe tricuspid regurgitation (TR) with a peak TR velocity (TRV) of 401.2 cm/seconds and estimated pulmonary artery systolic pressure (PASP) >80 mmHg. There was a moderate-sized pericardial effusion without signs of tamponade. Trans-oesophageal echocardiography (TEE) was done and revealed a positive agitated saline contrast study (2+ after 3–4 cardiac cycles) and ensured the inter-atrial shunting via ASD. Right heart catheterization (RHC) was done and revealed: mPAP: 53 mmHg, PCWP: 6 mmHg, PVR: 17 WU, cardiac output (CO): 3.13 L/minutes, and negative vasoreactivity. Chest computed tomography (CT scan) was done and revealed bilateral ground-glass opacities with smooth septal thickening, enlarged pulmonary trunk, and mediastinal lymphadenopathy. The patient was diagnosed to have primary pre-capillary PH after the exclusion of secondary aetiologies. Together with the radiological features of bilateral ground glass appearance and mediastinal lymphadenopathy, the probability of PVOD was raised. The patient received diuretics only, and pulmonary vasodilators were not prescribed for risk of deterioration until confirmation of diagnosis. A genetic study was planned but the patient traveled to her town and did not attend the appointment (Figures 1 and 2).

**Figure 3:**
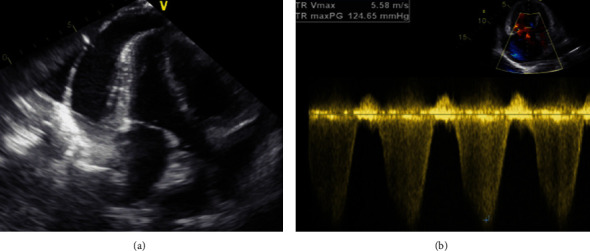
Transthoracic echocardiography revealed severe dilatation and systolic dysfunction of the right ventricle while normal left ventricle and a large pericardial effusion without tamponade (a). There was a severe TR with an estimated PASP >120 mmHg and a TRV 5.58 m/s (b).

After 2 months, she was referred to our tertiary care hospital with unstable hemodynamics, acute hypoxemic respiratory failure, and severe right-sided heart failure. She gave a history of furosemide and sildenafil therapy at a local hospital with progressive deterioration of her condition. Upon the second admission BP was 92/58 mmHg, HR was 134 bpm (sinus tachycardia), SPO2 72%, CVP was 22–28 cmH_2_O, and bilateral lower limb massive pitting oedema. Emergency hemodynamic and ventilator support was done using inotropes infusions and non-invasive and then invasive mechanical ventilation. Laboratory workup revealed: NT-proBNP 2600 (pg/mL) and normal other laboratory variables. A genetic study revealed the eukaryotic translation initiation factor 2 alpha kinase 4 (EIF2AK4) gene on chromosome 15, which was diagnostic to PVOD. Echocardiography revealed severe right ventricle dilatation with severely reduced function, a normal-sized left ventricle with EF of 50–55%. There was a severe TR with a peak TRV of 558 cm/seconds and an estimated PASP > 120 mmHg. There was a large-sized pericardial effusion without signs of tamponade (Figure 3).

**Figure 4:**
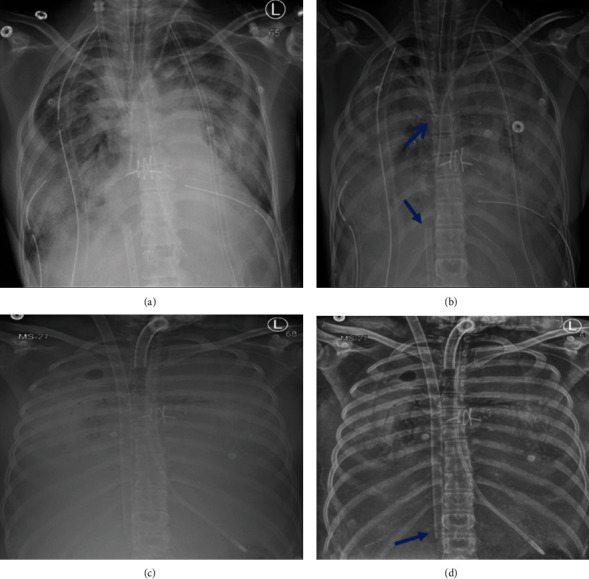
Post-lung transplant CXR with diffuse ground glass opacity and airspace consolidation (a) with jugular and femoral ECMO cannulas (blue arrows) in the line view (b). Complete opacification of both lungs occurred (c) with prolonged mechanical ventilation, tracheostomy, and VV-ECMO support via Avalon cannula (blue arrow) in line view (d).

Emergency peripheral veno-arterial ECMO (VA-ECMO) was inserted at the operating room due to small-sized femoral vessels as assessed with vascular ultrasound. A 20-French arterial cannula was inserted inside a Dacron graft that was inserted in the femoral artery via end to side approach. Jugular and femoral venous cannulas were inserted and connected to the drainage cannula of ECMO to achieve good venous drainage. An urgent workup for lung transplantation was done, and the patient was found to be highly sensitized. The protocol for crossmatching positive transplantation was used with plasmapheresis sessions, and immunosuppression then bilateral lung transplantation were done after 5 days of VA-ECMO support. Unfortunately, the patient developed acute graft rejection as diagnosed by transbronchial biopsy and required prolonged mechanical ventilation and ECMO was changed to veno-venous configuration (VV-ECMO; Figure 4).

**Figure 5:**
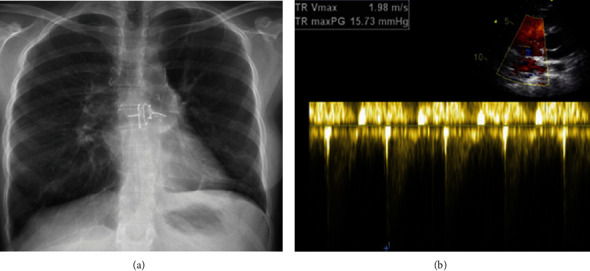
Predischarge CXR (a) revealed clear lung fields with a normal cardiac shadow. TTE (b) showed mild TR with a TRV of 1.98 m/s.

As the patient was fully awake with a stable hemodynamic profile and good multi-organ functions, the patient was listed again for transplantation. Redo bilateral lung transplantation was done, and the patient was successfully weaned off support with 4-month ECMO support and 6-month ventilatory support. Echocardiography was repeated and revealed normal size and contractility of both ventricles with mild TR. The patient was discharged with stable hemodynamics and SPO2% 98% on room air (Figure 5).

Immune suppression was maintained using tacrolimus, mycophenolate mofetil, and corticosteroid therapy. During the 4-year follow-up, the patient had recurrent chest infections and received antifungal and antiviral therapies. Recently, she had dyspnea with impaired PFTs and was diagnosed to have antibody-mediated chronic lung rejection, for which she was admitted to our hospital and received plasmapheresis sessions, pulse corticosteroid, and intravenous immunoglobulins therapy. Currently, she is alive with a stable hemodynamic profile and no oxygen dependency.

## 3. Discussion

Our patient is a young female without a significant medical history and presented with PH and right-sided heart failure. The initial diagnosis was idiopathic PAH after the exclusion of left-sided heart disease, autoimmune and connective tissue diseases, hematological disorders, and drugs and infections associated with PH. The RHC revealed pre-capillary PH, which was consistent with idiopathic PAH, but the PVR was high, and the vasoreactivity test was negative, which was against the diagnosis. The genetic and radiological studies and the deterioration after pulmonary artery vasodilators made help to reach the diagnosis of PVOD.

In PVOD, the pulmonary hemodynamic profile and worse outcome after vasodilator therapy are explained by the pathological features. PVOD is characterized by diffuse intimal fibrosis and obliteration of the pulmonary venules, engorgement of the alveolar capillaries, and occasionally angioproliferation occur giving a picture that mimics pulmonary capillary haemangiomatosis (PCH). Therefore, PVOD is associated mainly with post-capillary and capillary obliteration, whereas arterial affection is minimal compared with idiopathic PAH [7, 8]. During RHC, the PCWP is <15 mmHg due to the sparing of the large pulmonary veins, whereas the true pulmonary capillary and arterial pressures are increased [9]. The increased pulmonary capillary pressure cannot be directly measured but can be estimated from analysis of the decay curve of pulmonary artery pressure after balloon occlusion [10]. The increased pulmonary capillary pressure explains the development of life-threatening pulmonary oedema with pulmonary artery vasodilator therapy, which leads to capillary flooding. Even with vasoreactivity testing, patients with PVOD may develop acute pulmonary oedema [11, 12]. Very short-term vasodilatation with inhaled nitric oxide for 5–10 minutes during vasoreactivity testing was safe and did not produce pulmonary oedema during the catheterization of PVOD-confirmed patients [9].

Recently, the European Society of Cardiology/European Respiratory Society guidelines recommended combining clinical and radiological features with PFTs and genetic study as class IA to diagnose PAH with POVD/PCH suspicion and requesting biallelic EIF2AK4 mutations as class IA recommendation to confirm the diagnosis [1]. In addition, the current guidelines recommended (class IC) refer patients with POVD/PCH to lung transplant centers for evaluation [1]. A comprehensive echocardiographic study is a valuable tool to diagnose PH and right ventricle function with the estimation of PASP and TRV. In addition, echocardiography helps to diagnose the presence of right to left shunting and assessment of left-sided heart morphology and function [1, 13–15]. While CXR has a limited value in the diagnosis of PVOD unless there is pulmonary oedema, a high-resolution CT scan is a valuable tool with a triad of ground glass opacities, mediastinal lymphadenopathy, and interlobar septal thickening. [9, 16] However, the absence or existence of only one of the CT triads do not exclude the PVOD diagnosis [5]. Moreover, a CT scan helps to accurately assess the lung parenchyma and exclude group-3-PH. Right cardiac catheterization is the gold standard for the diagnosis and classification of PH, pre-operative assessment for lung transplantation candidates, and vasoreactivity testing before pulmonary vasodilator therapy [1, 9, 17].

When our patient presented with pulmonary oedema and cardiogenic shock, emergency peripheral VA-ECMO support was started after the failure of stabilization with invasive ventilation and inotropic support. Emergency lung transplantation was arranged to avoid the potential complications associated with emergent VA-ECMO, especially with a young female with a low BMI and small-sized vessels [18–21]. Pericardiocentesis was not tried despite the presence of a large-sized pericardial effusion and hemodynamic instability due to the presence of severe PH and the possibility of further deterioration and development of pericardial decompression syndrome (PDS) [22–24]. PDS is a paradoxical deterioration of hemodynamics after therapeutic pericardiocentesis [23].

According to the recent consensus report of the International Society for Heart and Lung Transplantation (ISHLT), lung transplantation should be considered early in patients with PAH before the deterioration of cardiopulmonary functions to have enough time for appropriate evaluation and suitable donor selection [25]. The ISHLT approved the use of ECMO in patients with respiratory failure or right ventricular failure as a bridge to transplant in experienced centers [25]. After ECMO support of our patient and lug transplantation, the patient developed acute rejection with primary graft dysfunction (PGD), which is associated with early mortality [26]. The PGD usually occurs within the first few days of lung transplant and is related to lung ischemia and reperfusion injury, and acute rejection is suspected in case of no improvement of lung oedema [26, 27]. Despite the graft failure and repeated infections, continuous support with VV-ECMO was maintained till redo lung transplantation was done, which had a successful outcome.

Finally, the diagnosis of PVOD required a high index of suspicion and to be differentiated from idiopathic PAH to avoid using the pulmonary vasodilators and early evaluation for lung transplantation should be planned before clinical deterioration. The use of ECMO support as a bridge to lung transplantation was successful during the development of PGD until the redo transplantation.

## 4. Conclusions

Clinical recognition of PVOD is crucial due to its challenging diagnosis, need for genetic study, rapid deterioration with pulmonary vasodilators, and bad prognosis. Lung transplantation is the definitive treatment for eligible candidates.

## Data Availability

Data supporting this research article are available from the corresponding author or first author upon reasonable request.

## Abbreviations

PH: Pulmonary hypertension
PAH: Pulmonary arterial hypertension
PASP: Pulmonary artery systolic pressure
PCWP: Pulmonary capillary wedge pressure
PVOD: Pulmonary veno-occlusive disease
PVR: Pulmonary vascular resistance
PDS: Pericardial decompression syndrome
PFTs: Pulmonary function tests
ERS: European Respiratory Society
ESC: European Society of Cardiology
TRV: Tricuspid regurgitation velocity
TEE: Trans-oesophageal echocardiography
TTE: Transthoracic echocardiography
NT-proBNP: N-terminal pro B-type natriuretic peptide
ANA: Antinuclear antibody
ANCA: Anti-neutrophil cytoplasmic autoantibody
Anti-SSA: Anti-Sjögren's syndrome-related antigen A autoantibodies
Anti-SSB: Anti-SS-antigen B autoantibodies
BMI: Body mass index
CO: Cardiac output
CT: Computed tomography
RHC: Right heart catheterization
ECMO: Extracorporeal membrane oxygenation
EIF2AK4: Eukaryotic translation initiation factor 2 alpha kinase 4
WU: Wood unit
ISHLT: International Society for Heart and Lung Transplantation.
